# Case Report: Adrenal tuberculosis causing primary adrenal insufficiency: diagnosis by ultrasound-guided biopsy

**DOI:** 10.3389/fmed.2025.1622617

**Published:** 2025-11-20

**Authors:** Tianzhen Xiong, Rongchen Wang, Jiaojiao Zhou, Boyang Yu

**Affiliations:** Division of Ultrasound, West China Hospital, Sichuan University, Chengdu, Sichuan, China

**Keywords:** adrenal tuberculosis, primary adrenal insufficiency, ultrasound-guided biopsy, ultrasound, ultrasound intervention

## Abstract

Adrenal tuberculosis, though a rare extrapulmonary manifestation of *Mycobacterium tuberculosis* infection, remains a significant etiology of primary adrenal insufficiency in tuberculosis-endemic regions. We present a case of a 38-year-old asymptomatic female patient incidentally found to have bilateral adrenal nodules with calcifications during routine health screening. Laboratory tests revealed significantly elevated adrenocorticotropic hormone levels and low plasma cortisol, while imaging studies showed bilateral adrenal masses. Despite anatomically challenging access, an ultrasound-guided core needle biopsy was successfully performed. Histopathological analysis identified caseating granulomatous inflammation, and the positive T-SPOT. TB assay confirmed the diagnosis of adrenal tuberculosis. This case illustrates an innovative biopsy approach for anatomically complex adrenal lesions, underscoring the diagnostic utility of image-guided techniques in rare adrenal disorders.

## Introduction

Primary adrenal insufficiency (PAI) is an endocrine disorder caused by adrenal cortex dysfunction, characterized by insufficient secretion of cortisol and aldosterone (1). Autoimmune diseases are the predominant etiology of PAI. In developed nations, autoimmune adrenalitis is the leading cause. However, in regions with a high incidence of tuberculosis, adrenal tuberculosis remains a significant cause (2, 3). In elderly and immunocompromised patients, beyond mycobacterium tuberculosis, other pathogens can also disrupt the bilateral adrenal cortical architecture, leading to functional failure. Fungal infections such as histoplasmosis, as well as other deep-seated mycoses-including par coccidioidomycosis and blastomycosis-may disseminate hematogenously to the adrenal glands and cause similar patterns of adrenal destruction (4). The opportunistic infectious etiologies of PAI are primarily cytomegalovirus and mycobacterium avium complex. Non-infectious causes encompass autoimmune adrenalitis, bilateral adrenal hemorrhage or infarction, and neoplastic diseases (e.g., metastatic tumors, lymphoma) (5).

## Case report

A 38-year-old female patient presented to our hospital for further diagnosis and treatment after the discovery of bilateral adrenal nodules during a routine physical examination. She exhibited mild pigmentation over the interphalangeal joints of her hands and knees, yet she did not present with typical symptoms of tuberculosis, such as low-grade fever, night sweats, or cough. Three months ago, outpatient testing revealed elevated adrenocorticotropic hormone (ACTH) levels at 577.10 ng/L (reference range, 5.00–78.00 ng/L) and decreased plasma cortisol at 161.00 nmol/L (reference range, 133.0–537.0 nmol/L), consistent with PAI. Five years ago, she was hospitalized for a “chest shadow”; however, tuberculosis was not diagnosed, and no anti-tuberculosis treatment was administered. She denied any history of glucocorticoid use and has no family history of hereditary diseases.

**Figure 1:**
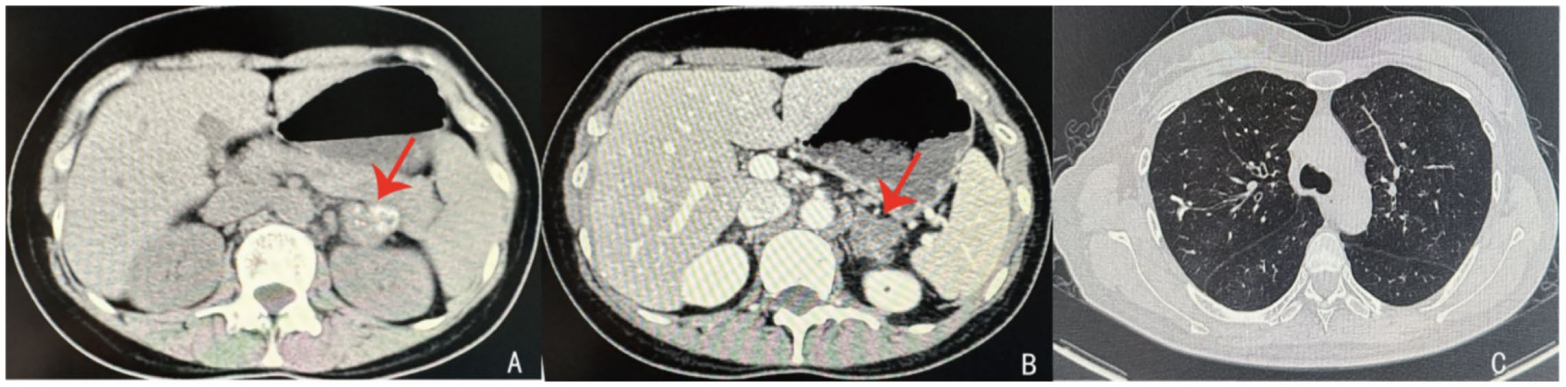
Left adrenal nodule with calcification (**A,B**; red arrow) and old tuberculosis lesions in both lungs and pleura **(C)**.

**Figure 2:**
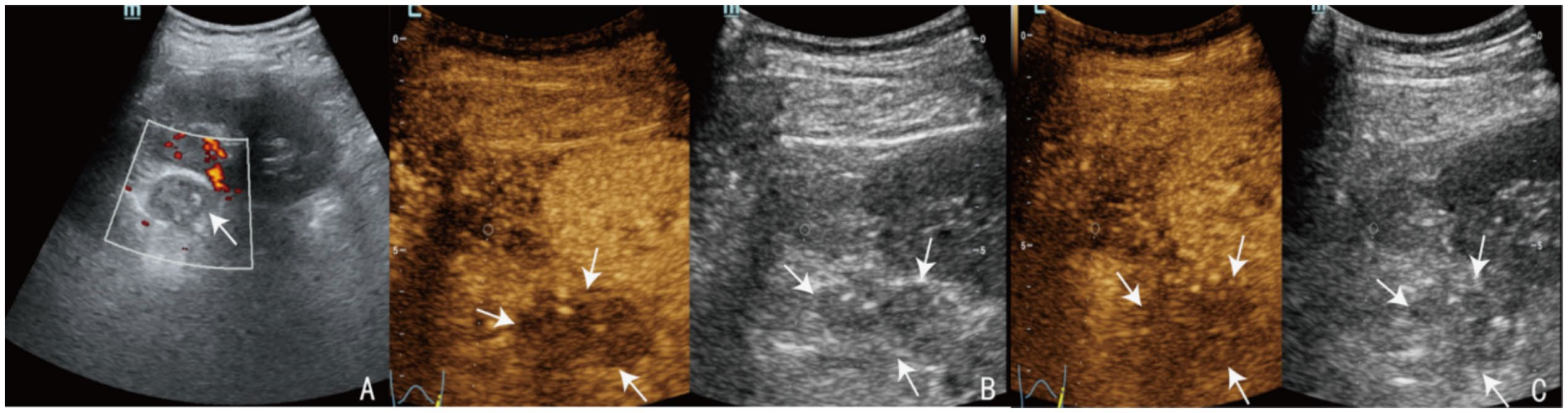
Two-dimensional ultrasound and contrast-enhanced ultrasound images of left adrenal tuberculosis. A hypoechoic nodule with calcification measuring approximately 2.7 × 2.2 cm was observed in the left adrenal region **(A)**. Contrast-enhanced ultrasound demonstrates heterogeneous hypoenhancement of the nodule during both the corticomedullary and delayed phases **(B,C)** (the arrow indicates the lesion).

**Figure 3:**
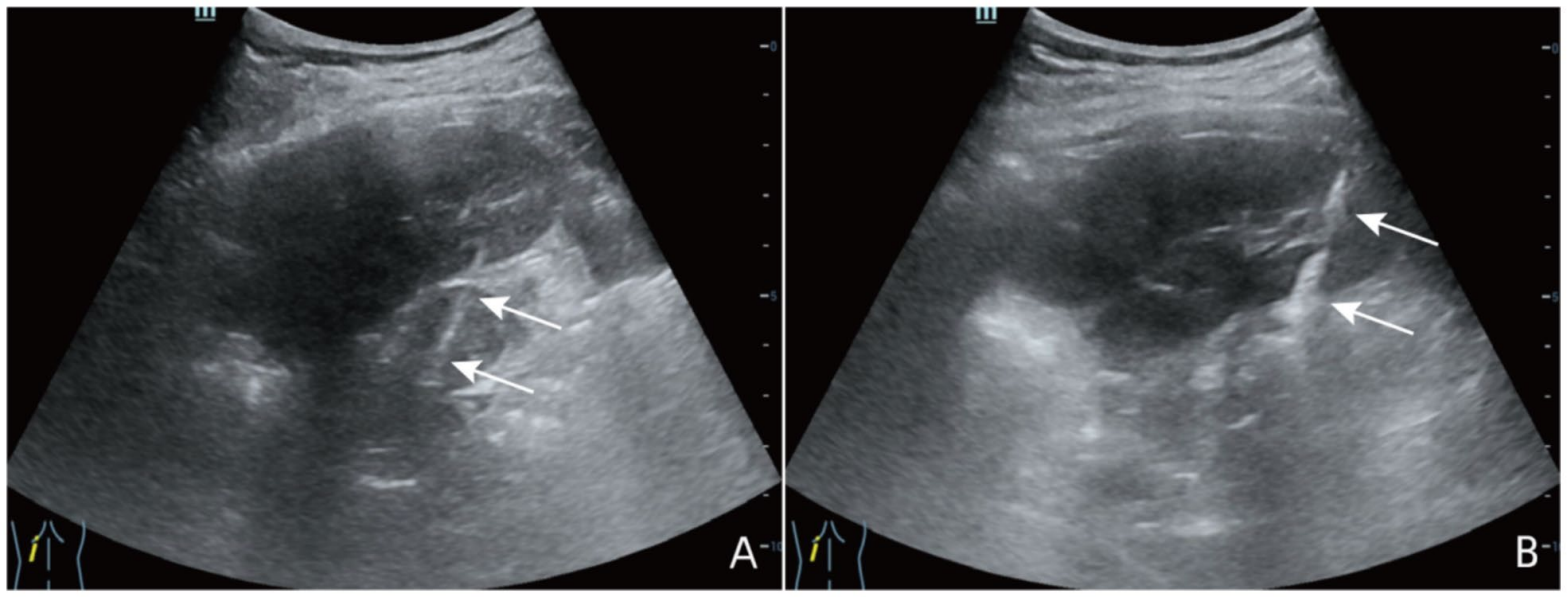
The process of percutaneous biopsy. **(A)** The arrow indicated the biopsy needle passing through the parenchyma of the left kidney to reach the left adrenal mass. **(B)** The arrow indicated the gelatin sponge used to occlude the needle tract.

**Figure 4:**
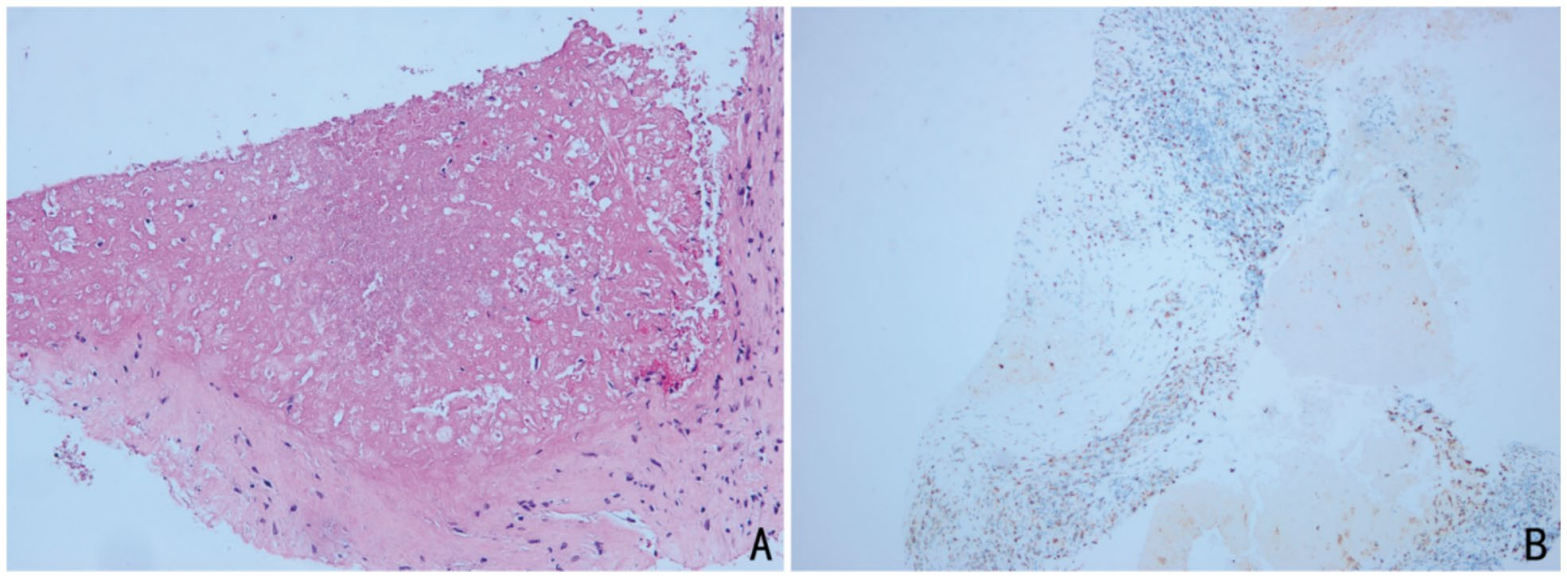
Pathological results: **(A)** HE stains, ×200: Granulomatous inflammation with necrosis; **(B)** Immunohistochemistry: Tissue cells CD68PG-M1 (+).

Laboratory results at admission showed significantly elevated ACTH (365.80 ng/L) and decreased plasma cortisol (139.00 nmol/L), along with low fasting blood glucose and sodium levels, indicating adrenal insufficiency. Meanwhile, renin-angiotensin-aldosterone ratio (ARR 0.09) was consistent with adrenal insufficiency and the 25-hydroxyvitamin D was low (20.6 nmol/L; reference range, 47.7-144 nmol/L). The T-SPOT. TB test was positive, further supporting the diagnosis. The T-SPOT. TB test for tuberculosis was positive, further supporting the diagnosis of tuberculosis. Computed tomography (CT) scans revealed bilateral adrenal nodules with calcification and old tuberculosis lesions in both lungs and the pleura (Figure 1). Ultrasonography showed hypoechoic nodules with calcification in both adrenal glands, the largest of which was located on the left side, measuring approximately 2.7 × 2.2 cm in size. Contrast enhanced ultrasound demonstrated low intensity enhancement during both corticomedullary and delayed phases (Figure 2). Ultrasound-guided biopsy of the adrenal lesions was performed. Due to the thin strip shape, the right adrenal mass was unsuitable for biopsy. The left adrenal mass was slightly larger but surrounded by the pancreas, spleen, and kidney, making the biopsy path anatomically complex and challenging. After a multidisciplinary discussion, percutaneous biopsy by a route passing the left kidney under ultrasound guidance was designed. This route was chosen to avoid puncturing the spleen or pancreas. Under guidance from a Color Doppler Ultrasound System (Resona R9, Shenzhen Mindray Bio-Medical Electronics Co., Ltd., China), we used a 17G coaxial needle (C1816B, 17 g × 13.0 cm, Bard Peripheral Vascular, Inc) to establish a biopsy tract. An 18G disposable biopsy needle (N1816, 18 g × 16 cm, Bard Peripheral Vascular, Inc) was then advanced through this coaxial system. And the biopsy path was then sealed with gelatin sponge (Gelfoam 560, 560 μm-710 μm, Hangzhou Alicon Pharmaceutical Co., Ltd) to reduce the risk of bleeding and contamination (Figure 3). Pathological examination revealed granulomatous inflammation with necrosis, and immunohistochemical staining demonstrated positive CD68PG-M1, negative SMA and S100 (Figure 4). Special staining, including acid-fast bacilli and silver staining, was negative. *Mycobacterium tuberculosis* qPCR analysis detected DNA fragments suggesting the presence of tuberculosis bacteria, confirming the diagnosis of adrenal tuberculosis. The medication regimen for the patient was as follows: For PAI, hydrocortisone acetate tablets (20 mg once daily) were administered, with dosage adjustments as clinically indicated, to maintain adrenal function. For adrenal tuberculosis, a first-line anti-tuberculosis regimen was prescribed: isoniazid (300 mg once daily), rifapentine (450 mg twice weekly), ethambutol hydrochloride (0.75 g once daily), and levofloxacin (0.5 g once daily). The follow-up included regular abdominal ultrasound and chest CT, along with monitoring of hormone levels for dosage adjustments. According to the results of the five follow-up visits, the patient’s symptoms have improved, hormone levels were gradually stabilizing, and the tuberculosis treatment regimen has shown a good response.

## Discussion

In developed countries, the predominant cause of PAI is autoimmune adrenalitis, characterized by the presence of anti-21-hydroxylase antibodies, a type of autoimmune disease (6, 7). In contrast, in developing countries, particularly in regions with a high prevalence of tuberculosis, tuberculosis remains one of the major causes of PAI. Adrenal tuberculosis is a rare form of extrapulmonary tuberculosis, presenting as a chronic, destructive inflammatory process that induces irreversible adrenal damage and loss of function (6). Adrenal tuberculosis, more commonly seen in young and middle-aged men, often involves both adrenal glands and presents with symptoms such as skin pigmentation, weight loss, fatigue, and anorexia (8). PAI is rare in clinical practice and its presentation lacks specificity. Furthermore, the imaging features of various adrenal diseases often overlap, making PAI diagnostically challenging and prone to misdiagnosis. Accurate identification of the cause is crucial for appropriate treatment. Studies have reported that about 68% of adrenal tuberculosis cases are misdiagnosed due to atypical symptoms (9). Undiagnosed adrenal tuberculosis is a potentially life-threatening condition, as adrenal crisis may occur during physiological stress in patients with bilateral adrenal involvement (10, 11). Early etiological identification is critical to guide targeted therapy.

Patients with pulmonary tuberculosis may develop adrenal enlargement even without direct adrenal infection, likely due to systemic inflammatory responses and chronic stress (4). Typically, patients with active or recent infection (within the last 2 years) present with bilateral adrenal enlargement, often accompanied by central necrosis and peripheral enhancement, a combination which represents an important radiological feature of adrenal tuberculosis (12). As the disease progresses to the chronic phase, enlarged adrenals may shrink or return to normal size and shape due to fibrosis and calcification (13). In this case, the patient had a prolonged course of illness, with bilateral adrenal masses on imaging.

When bilateral adrenal lesions occur simultaneously, it is important to differentiate them from adrenal tumors, particularly adrenal metastases, and lymphoma. Adrenal metastases are usually bilateral and associated with a known primary malignancy, rarely causing PAI (14). On contrast-enhanced imaging, adrenal metastases typically exhibit peripheral hyper-enhancement with rapid washout in the delayed phase. Adrenal abscess, typically arising as a secondary infection resulting from hematogenous dissemination, commonly manifests with clinical features such as fever, abdominal pain, and weight loss. On contrast-enhanced CT, the condition is characterized by bilateral adrenal enlargement featuring central hypodense areas (indicative of necrosis or liquefaction) and peripheral rim enhancement and calcifications may be observed in later stages (15). Adrenal lymphoma, though rare, typically appears as large, bilateral hypoechoic masses with more uniform enhancement on contrast imaging (16). Other conditions that need to be considered include pheochromocytoma, adrenal adenoma, myelolipoma, and adrenal hemorrhage.

While imaging plays a crucial role not only in the early detection of adrenal lesions but also in providing initial differential diagnostic information about the nature of the lesions, definitive diagnosis still relies on histopathological examination. Ultrasound-guided biopsy, a precise and minimally invasive diagnostic tool, is especially useful for lesions in anatomically complex locations. This technique allows for real-time imaging-guided tissue acquisition, minimizing the risk of injury to vital blood vessels and organs, thereby providing a reliable basis for diagnosis. Due to the unique anatomical position of the adrenal glands, most diagnoses of PAI caused by adrenal tuberculosis have primarily relied on imaging and laboratory results (17). Current guidelines recommend that core needle biopsy may be performed for adrenal lesions larger than 4 cm (18). In this case, the left adrenal mass was 2.7 cm in size, located anterior to the left kidney, and closely surrounded by the pancreas and spleen, making the biopsy path anatomically challenging. Ultimately, the biopsy was performed via the left kidney path under ultrasound guidance. Notably, after tissue collection, gelatin sponge was used through the coaxial needle to embolize the needle tract to reduce bleeding risk and contamination. Compared to CT-guided adrenal biopsy, ultrasound guidance demonstrates distinct advantages, including the absence of ionizing radiation, real-time imaging capability, procedural convenience, and higher cost-effectiveness, particularly when adequate target visualization and a safe needle trajectory can be achieved. However, ultrasound guidance is limited in situations where a secure puncture path is unattainable, such as when traversal of the renal collecting system is unavoidable or when intervening organs (e.g., bowel) impede access. Furthermore, in cases involving obese patients, extensive bowel gas, or small deeply located lesions, CT guidance emerges as an essential and reliable alternative owing to its superior tissue contrast resolution and consistent imaging performance unimpeded by acoustic limitations.

This case emphasizes an innovative technical approach to biopsy for adrenal tuberculosis in complex anatomical locations and serves as a reminder for clinicians in tuberculosis-endemic regions to maintain a high index of suspicion for adrenal tuberculosis in patients presenting with PAI. By combining contrast-enhanced ultrasound, biopsy, and molecular diagnostic techniques, early diagnosis of adrenal tuberculosis clinicians can achieve, allowing for prompt treatment and improved patient outcomes. The diagnostic and treatment experience in this case provides valuable reference for similar cases and offers a basis for the development of more efficient diagnostic and therapeutic strategies.

## Conclusion

As a rare yet clinically significant etiology of PAI, adrenal tuberculosis warrants heightened clinical vigilance in TB-endemic areas. This case demonstrates that ultrasound-guided biopsy serves as a definitive diagnostic tool while providing critical technical insights for managing anatomically challenging lesions. Given its minimally invasive nature and superior diagnostic yield, ultrasound-guided adrenal biopsy should be regarded as the diagnostic modality of choice for suspected adrenal tuberculosis.

## Data Availability

The original contributions presented in the study are included in the article/supplementary material, further inquiries can be directed to the corresponding author/s.
